# Impact of Autoclaving on the Dimensional Stability of 3D-Printed Surgical Guides for Aesthetic Crown Lengthening

**DOI:** 10.3390/jfb16080284

**Published:** 2025-08-02

**Authors:** Albert González-Barnadas, Anna Ribas-Garcia, Adrià Jorba-García, Rui Figueiredo, Eduard Valmaseda-Castellón, Octavi Camps-Font

**Affiliations:** 1 Department of Dentistry, Faculty of Medicine and Health Sciences, Universitat de Barcelona, 08907 Barcelona, Spain; agonzalezbarnadas@ub.edu (A.G.-B.); aribasga40@alumnes.ub.edu (A.R.-G.); eduardvalmaseda@ub.edu (E.V.-C.); ocamps@ub.edu (O.C.-F.); 2 Dental and Maxillofacial Pathology and Therapeutics Research Group, IDIBELL Research Institute, 08908 Barcelona, Spain

**Keywords:** aesthetic crown lengthening, surgical guide, sterilization, resin, 3D printer

## Abstract

The aim of this study was to evaluate the impact of autoclaving on the dimensional stability of surgical guides (SGs) for aesthetic crown lengthening (ACL) using different resins/printing methods. Fifty SGs for ACL were printed using five different resin/printer combinations (FL, SR, ND, KS and VC). All the SGs were scanned before (T0) and after (T1) sterilization. Autoclaving was conducted at 134 °C during 4 min. The STL files of each SG at T0 and T1 were compared with the original design (TR). Dimensional stability was measured using trueness and precision. Deviations from TR to T1 were calculated in the three space axes and by measuring the area between three reference landmarks. At T0, the FL group showed the best trueness and precision, while the SR group performed significantly worse than the other groups. At T1, all the groups except VC exhibited significant dimensional alterations compared with T0. Also, VC showed the best trueness and precision values. All the groups had a significant deviation in at least one space axis, while only the SR group exhibited significant variations from T1 to TR in the area between the reference landmarks. Most of the evaluated resin/3D printer combinations suffered significant dimensional alterations after autoclaving.

## 1. Introduction

In recent years, “computer aided surgery” has earned a lot of significance in dentistry, specially within the field of implantology. Extensive evidence supports the effectiveness of guided surgery, both static or dynamic, in improving the precision of implant placement in many different scenarios [1,2]. Other dental surgical procedures have taken advantage of computer-aided technology, such as canine fenestration [3], tooth autotransplantation [4], corticotomies [5] or aesthetic crown lengthening (ACL) [6]. Specifically, in the latter procedure, many recent publications have described the application of surgical guides (SGs) to enhance precision in gingivectomy and ostectomy around the involved teeth [6,7,8,9,10,11,12,13]. While some of the authors recommend using two different SGs for each step [7,9], most prefer to use one single “dual” SG with landmarks guiding both gingivectomy and ostectomy [6,8,10,11,12,13]. A recent case-series [14] described high accuracy and stability over six months using a dual SG in patients diagnosed with altered passive eruption (APE). In a randomized–controlled clinical trial, the authors did not find a significant improvement in terms of precision when comparing guided and non-guided ACL [10]. However, they noted a significant reduction in terms of surgical time when using SG in comparison to freehand surgery. It may also be argued that computer-aided surgery in such precise procedures may increase predictability when novice professionals are involved.

Advancements in computer-aided design and computer-aided manufacturing (CAD-CAM) technology have made the design and manufacturing of SGs more straightforward to the majority of the clinicians. While in the past the fabrication of such guides was mainly performed with subtractive methods (SMs), now additive methods (AMs) seem to be more cost-effective, since they use less material, are faster and require less infrastructure for its manufacturing [15]. Additive methods, commonly known as 3D printing, involve many different technologies used to create the designed object by using a layer-by-layer construction from a base of a powder, liquid or solid filament/sheet material [16,17]. Within the manufacturing of SGs, one of the most widely employed AM methods is *vat-polymerization*, in which a liquid resin is selectively polymerized by a light source. Vat-polymerization methods include stereolithography (SLA), digital light processing (DLP), liquid crystal display (LCD) and continuous liquid interface production (CLIP) printing.

Recent in vitro studies suggest that the choice of material, manufacturing process, storage conditions and sterilization methods can influence SG dimensional stability, particularly for implant SGs [18,19,20,21,22]. While some authors report superior performance of SM compared to AM [22], others found no statistically significant differences [15]. According to Rouzé l’Azit et al. [18], among AM, vat-polymerization SLA and DLP printing showed a higher trueness and precision than other methods. Also, in a recent study, the authors suggested that the accuracy between these two methods depended on the area of the SG that was evaluated [19]. While DLP printing showed a higher overall accuracy, SLA printing showed better results in the regions of interest (ROIs) at the occlusal zone and guide hole.

Ntovas et al. [20] found out that storage conditions of SGs with direct or indirect exposure to daylight could have an impact on their dimensional stability in the short term (up to 3 months). Moreover, the sterilization method may also have an impact on the dimensional stability of the SG [21,23,24,25]. Li et al. [21] evaluated the effect of many disinfection/sterilization methods, including hydrogen peroxide, glutaraldehyde, autoclaving, plasma sterilization and iodophor disinfection. All the methods had a certain impact on the size of the SG, but the plasma sterilization group showed less deformation than the other groups. Considering that the most widely employed sterilization method in the dental clinics is autoclaving, many studies have focused on it. It has been reported that, either with SLA [24] or DLP [23] resin SGs, autoclaving at 121 °C during 20 min has an impact of SG stability. When comparing this cycle with one at 134° for 5.5 min, Hüfner et al. [25] found that most of the resin SLA, DLP and LCD SGs suffered deformations. Only one DLP guide showed no dimensional alterations independently of the autoclaving cycle.

It is uncertain whether these dimensional alterations of the SG are clinically relevant. All this knowledge is based on in vitro studies focused on dental implant SGs and consequently it is important to evaluate the impact of sterilization processes on the dimensions of SGs used for other purposes. For instance, SGs employed for ACL may be thinner in certain areas than those used for implant surgery, and this could affect their dimensional stability to a higher degree when subjected to an autoclaving process. Also, considering the aesthetic nature of ACL, achieving the highest level of accuracy is crucial when using SGs for this purpose. Therefore, the aim of this study was to evaluate the impact of autoclaving on the dimensional stability of SGs for ACL surgery manufactured using different resins/printing methods.

## 2. Materials and Methods

An in vitro study was conducted in order to evaluate the impact of autoclaving on the dimensional stability of 3D-printed surgical guides using 5 different resin/3D printer combinations. The protocol was developed in accordance with the tenets of the Helsinki Declaration and was approved by the Ethics Committee (CEIm) (Protocol number 2023-040-1).

**Figure 1:**
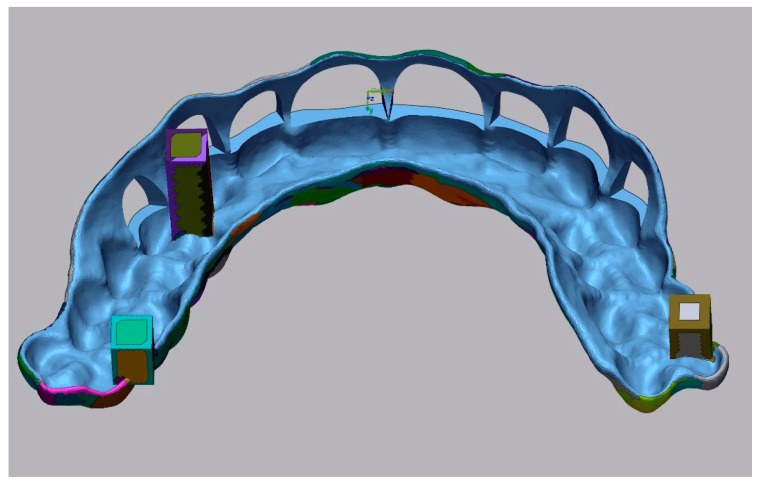
Design of the original SG at the segmented intaglio surface (ROI).

After obtaining proper informed consent, an SG was designed for clinical purposes to perform an ACL surgery in a patient diagnosed with APE type 1B, as described in a previous case-series study [14]. The Digital Imaging and Communication in Medicine (DICOM) files obtained from the cone-beam computed tomography (CBCT) (NewTom GiANO HR; Quantitative Radiology, Verona, Italy; 0.2 mm/voxel, 110 kV, 64.6 mAs, 4.3 s) scan of the patient were converted to Standard Tessellation Language (STL) format and then superimposed with STL files acquired from the intraoral scan (Trios 3; 3Shape, Copenhagen, Denmark) using Exocad DentalCAD software (3.2 Elefsina, Exocad GmbH, Darmstadt, Germany). With this design software, the CEJ level of each tooth to be treated was marked to guide the gingivectomy incision line. In addition, a second line was positioned 3 mm apical to the cementoenamel junction (CEJ) line to guide the bone resection. This virtual design was performed with a thickness of 2 mm and a 0.03 mm guide-to-teeth offset. In order to measure dimensional stability, 3 square-shaped structures were added on the intaglio surface of the SG at the level of right first premolar (DE) and both right (AF) and left (BC) second molars as reference landmarks (Figure 1).

Sample size was calculated using the software G*Power v.3.1.3 (Heinrich-Heine Universität, Dusseldorf, Germany). Considering a minimum expected effect size (*f*) of 1, 5 study groups and 2 timepoints, 10 specimens per group were required (repeated-measures analysis of variance, α = 0.05 and 1-β = 0.90). Following this calculation, 50 SG were printed using 5 different resin/3D printer combinations (10 for each experimental group). One group (FL) employed a stereolithographic (SLA) printer, two groups (SR and ND) employed a digital light processing (DLP) printer and the other two groups (KS and VC) employed a liquid crystal display (LCD) printer. All printers were calibrated according to the manufacturer’s instructions. The details on the experimental groups in terms of the type of resin, printing method and post-processing are described in Table 1 and Table A1.

Once printed, all SGs were codified and scanned using an E4 laboratory scanner (3Shape, Copenhagen, Denmark) with a precision of 4 μm and converted into a STL model at baseline (T0). Before the first scanning, all SGs were coated with a thin and uniform layer of scanning spray (Renfer Scanspray, Renfert Corp, Hilzingen, Germany). Afterwards, all SGs were subjected to a steam autoclaving process at 134 °C and 2.1 bars during 4 min (M20-B plus (Matachana, Barcelona, Spain)). After sterilization (T1), all SGs were re-scanned using the same scanning process to obtain a new STL model.

**Figure 2:**
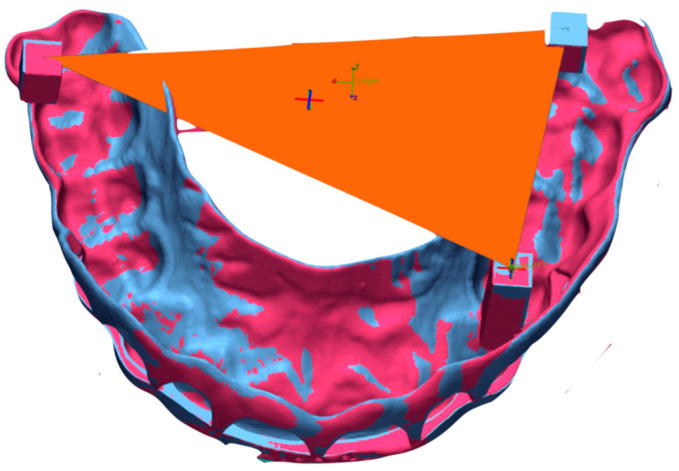
Measurement of the area between the three square-shaped structures.

All the STL files were exported to Geomagic Control X software version 2023.0.1 (3Dsystems, Rock Hill, SC, USA). The original CAD design of the SG was employed as a master specimen (TR), and segmentation was performed to define a region of interest (ROI) at the intaglio surface of the SG. Dimensional stability of the SGs was evaluated by aligning the STL files of each SG at T0 and T1 with the master SG (TR) with the best fit algorithm, using a discrepancy of 1000 μm and a tolerance range of ± 100 μm. Three-dimensional (3D) deviation was evaluated using trueness and precision values evaluated at the ROI of the SG. According to the International Normalization Organization (ISO) [26], trueness is defined as the closeness of agreement between test results and the real value, and its measurement is performed by using the *root mean square* (RMS). Precision is defined as the closeness of agreement between independent test results, and it is measured by using the *standard deviation* (SD). Axis deviations between the master surgical guide (TR) and each individual printed guide were calculated at the zenith point of a randomly selected arch to determine the directional components of deviation along the X, Y and Z axes. In addition, the surface area enclosed by the three square-shaped reference structures was measured and compared to that of the reference guide (TR: 712.73 mm^2^) to evaluate dimensional discrepancies between the guides (Figure 2).

Statistical analyses were conducted using R software version 4.5.0 (Development Core Team 2025). Descriptive statistics, including mean values, standard deviations and 95% confidence intervals (95%CIs) were calculated stratified by study group and time. To evaluate the effects of autoclaving (T0 or T1), printer/resin type (FL, SR, ND, KS or VC) and their interaction on RMS and SD values, a repeated-measures mixed-effects model was applied. Pairwise comparisons between groups were conducted for each printer/resin and timepoint. To evaluate directional deviations of the guide arch in sterilized samples along the X, Y and Z axes, as well as surface area differences relative to the CAD reference model, one-sample t-tests were conducted for both analyses. The level of significance was set at *p* < 0.05, using the Tukey correction for multiple comparisons. The assumptions underlying the statistical analyses were checked in all cases.

## 3. Results

All samples were treated without registering any deviation from the protocol.

### 3.1. Trueness and Precision Analysis

The repeated-measures linear mixed-effects model revealed significant main effects of sterilization (*F*_1,77_ = 100.91, *p* < 0.001), resin/printer combination (*F*_4,77_ = 24.41, *p* < 0.001) and their interaction (*F*_4,77_ = 10.360, *p* < 0.001) on trueness values (RMS). Similarly, for precision values (SD), the model showed significant effects of time (*F*_1_,_77_ = 104.16, *p* < 0.001), group (*F*_4,77_ = 25.04, *p* < 0.001) and the interaction between these two factors (*F*_4,77_ = 10.56, *p* < 0.001).

**Figure 3:**
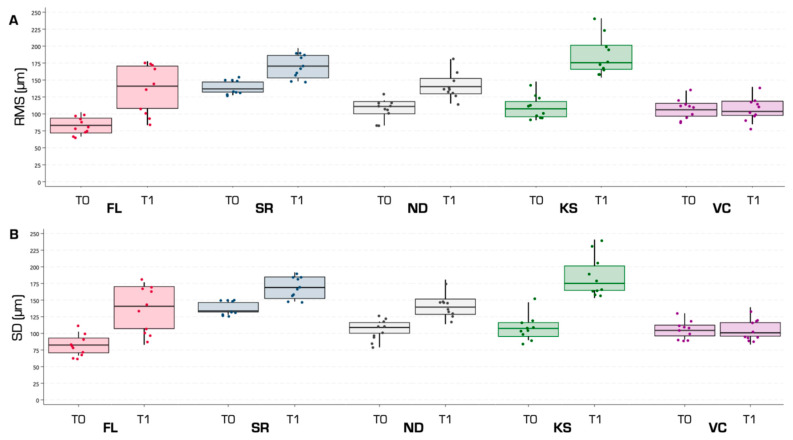
Trueness (**A**) and precision (**B**) of the experimental groups at T0 and T1. RMS: root mean square; SD: standard deviation; T0: timepoint before autoclaving; T1: timepoint after autoclaving; FL: Formlabs Form 3/Dental SG; SR: SprintRay Pro 95S/Surgical Guide 3; ND: NextDent 5100/NextDent SG; KS: Phrozen Mighty 4K/Keyguide; VC: Shining Accufab L4D/V Print SG.

**Figure 4:**
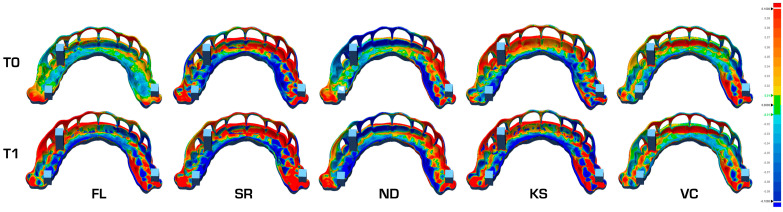
Color map of a representative SG of each group at T0 and T1. Warm colors (yellow to red) indicate the areas where the actual SG is larger than the reference guide, and cold colors (turquoise to blue) indicate the areas where the actual SG is smaller than the reference guide. T0: timepoint before autoclaving; T1: timepoint after autoclaving; FL: Formlabs Form 3/Dental SG; SR: SprintRay Pro 95S/Surgical Guide 3; ND: NextDent 5100/NextDent SG; KS: Phrozen Mighty 4K/Keyguide; VC: Shining Accufab L4D/V Print SG.

Analysis of trueness and precision within the ROI revealed statistically significant dimensional changes from T0 to T1 in all groups except VC (*p* < 0.05) (Figure 3, Table 2). A color-coded deviation map illustrating representative samples from each group at both timepoints is shown in Figure 4.

At baseline (T0), the SR group had a significantly greater dimensional change than the other groups (*p* < 0.05; Table 3), which did not show significant differences among them. The FL group demonstrated the highest accuracy, with significantly lower trueness values than all other groups and significantly higher precision compared to KS and SR.

Following sterilization (T1), the VC group showed the lowest discrepancy values, with statistically significant differences relative to all other groups (*p* < 0.05; Table 4). The FL and ND groups did not differ significantly from each other (RMS: *p* = 0.973; SD: *p* = 0.974), but both showed significantly better accuracy than SR and KS (*p* < 0.05). No statistically significant differences were found between SR and KS (RMS: *p* = 0.398; SD: *p* = 0.335).

### 3.2. Axis Deviations at the Zenith Point

All groups exhibited at least one statistically significant deviation along one of the three axes. Significant deviations in the Y axis between TR and T1 were observed in all groups (*p* < 0.05). For the X axis, all groups except ND showed significant changes over time. In the Z axis, only ND and FL did not demonstrate significant deviations, whereas the remaining groups exhibited statistically significant differences from TR to T1.

### 3.3. Area Variation

Surface area measurements of the three square-shaped structures at T1 are summarized in Table 5. Compared to TR, significant changes were observed only in the SR group (*p* < 0.001), whereas no statistically significant variations were detected in the remaining groups.

## 4. Discussion

The results of the present study suggest that most of the resin/3D printer combinations experience significant dimensional alterations when subjected to steam sterilization, considering that only one out of five tested groups did not suffer such significant variations.

To our knowledge, this is the first study addressing the dimensional stability of ACL-specific SGs after autoclaving. In contrast, most existing research focuses on SGs for dental implants [21,23,24,25,27] or orthodontic mini-implants [28]. Despite differences in the SG design, the results of the present study are consistent with prior findings [21,23,25,28], showing that autoclaving can variably affect the dimensional stability of SGs.

The clinical relevance of the observed deviations should be cautiously discussed. These deviations in most of the groups were below 0.1 mm, which may raise doubts regarding their clinical significance. However, all the groups suffered significant dimensional alterations in the zenith position along the *Y* axis, which is linked to vertical positioning of incisions and ostectomies and thus represents the most relevant axis from an esthetic point of view. Specifically, in the SR group, a deviation of 0.32 mm was recorded along the *Y* axis, more than three times the magnitude of the discrepancies observed in the other groups, which ranged from 0.06 to 0.13 mm. Deviations along the *X* axis may also be clinically relevant as they could displace the zenith point mesially or distally in relation to the planned position. The impact of these deviations observed at the target site might be aggravated by the deviations observed throughout the ROI and within the area compressed between the reference landmarks, potentially compromising the proper fitting of the SG. The cumulative impact of these discrepancies and their clinical significance should be further investigated in prospective clinical studies, ideally through randomized controlled trials. Additionally, the definition of a clinically acceptable threshold would be advisable to objectively assess the relevance of the deviations from the original planning.

In the present study, the only group whose trueness and precision were not significantly affected by the autoclaving process was the VC group, composed of SGs manufactured by the combination of V Print SG resin (Voco GmbH, Cuxhaven, Germany) and the Shining Accufab L4D 3D printer (Shining 3D, Hangzhou, China). This group also demonstrated the best trueness and precision values at T1 when compared to the other groups. Although axial measurements in VC groups exhibited significant alterations at T1 compared with the reference SG (TR), these variations remained among the lowest compared with the other groups, especially at the X and Y axes, where the deviations did not exceed 0.1 mm. Also, the area between the square-shaped structures remained stable when compared with the reference SG (TR). The same resin was used in the study performed by Yazigui et al. [23], which also showed the lowest discrepancies after steam sterilization among six tested resins. Also, Pranno et al. [29] found that “test bodies” printed using Shining Accufab L4D (Shining 3D, Hangzhou, China) exhibited fewer dimensional changes after autoclaving compared to those printed with two other LCD 3D printers.

Regarding ND and FL groups, similar trueness and precision values have been observed at T1, showing no statistically significant differences between them. Also, these groups were the only ones that did not exhibit significant variations at some space axis at this timepoint. While both showed significant variations at the Y axis, FL did not suffer significant alterations at the Z axis, and ND did not suffer significant alterations at the X and Z axis, reinforcing the behavioral closeness observed between both groups at the trueness and precision analysis. Hüfner et al. [25] studied the effect of two autoclave cycles (121 °C, 1 bar, 20.5 min and 134 °C, 2 bar, 5.5 min) in the dimensional stability of SGs manufactured using five resin/3D printer combinations, including the FL and ND groups assessed in this study. Their findings also indicated dimensional alterations post-sterilization, regardless of the autoclave cycle. Under similar conditions (134 °C, 5.5 min), FL and ND groups showed comparable behavior, with no statistically significant differences between them, which is consistent with our findings.

The worst trueness and precision values after sterilization were observed in SR and KS groups, which also showed significant alterations at the three space axes. The only group exhibiting significant variations from TR to T1 at the area measurements was SR. These alterations may be interpreted as a result of a significant contraction on the flanks of the SGs, which might have an impact on its proper fitting.

Regarding manufacturing accuracy before sterilization, the present study found that the FL group exhibited significantly better trueness and precision over the ROI at T0 compared to other groups, followed by ND, VC and KS, while SR performed the worst. These findings are in agreement with those of Morón-Conejo et al. [19], who also reported the highest accuracy for the FL group at the ROI. Furthermore, ND and VC showed comparable accuracy, with no significant differences between them, even though the VC group in that study used a different printer (VOCO SolFlex 170 SPM (Voco GmbH, Cuxhaven, Germany)). In relation to the printing methods, the only SLA printer that has been evaluated has achieved the highest accuracy among the tested groups. However, the impact of DLP and LCD printing methods on trueness and precision at manufacturing seems to be less important than the specific resin/3D printer combination, since significant differences were found between both DLP 3D printers (SR vs. ND), but no statistically significant differences were found between a DLP 3D printer and both LCD 3D printers (ND vs. KS and ND vs. VC). Vara et al. [30] observed significant differences in the accuracy of SG printing using the same DLP 3D printer with two different resins, which highlights the importance of the specific resin/3D printer combination. Thus, careful interpretation of the results is advised since using the same printer with a different resin, and vice versa, may affect the performance of the group.

The primary limitation of this study lies on its in vitro design, and therefore it would be advisable to evaluate the clinical impact of the observed discrepancies in more clinically oriented research. The absence of intraoral conditions (tissue contact, thermal fluctuation, humidity) could potentially influence the results. Additionally, the use of a single SG design based on one patient may introduce a design-specific bias, thus limiting the external validity of the results.

Given the dimensional impact of autoclaving on these SGs and the potential clinical relevance due to the aesthetic demands of the ACL procedure, alternative disinfection/sterilization methods should be considered. Some authors [21,27] suggest that plasma sterilization may have a low impact on dimensional stability of resin SG. However, its implementation in clinical dental settings remains limited due to higher equipment costs and logistical considerations. The present study employed a standard 134 °C, 2.1 bar and 4 min autoclave cycle, which is validated for all the resins and reflects the daily practice in our institution, replicating a clinically relevant and time-efficient protocol. A different type of cycle (121 °C, 1 bar, 20 min) may be employed, also being a common alternative, but few or no differences with 134 °C might be expected in terms of dimensional stability based on the results of some recent publications [25,27,28]. Alternative materials such as polyamide have been successfully employed to manufacture ACL SGs [14], and their dimensional stability after autoclaving should be tested to determine whether they outperform resin.

It is important to recognize that ACL SGs are structurally different from those used in implant surgery. They often feature thinner regions (around 2–3 mm thickness) in the gingivectomy/ostectomy arches, which may be more susceptible to deformation during sterilization. Therefore, further research is needed to evaluate alternative materials and sterilization methods for this specific type of SG, as well as their behavior after multiple sterilization cycles. Overall, while most tested resin–printer combinations exhibited dimensional changes after autoclaving, careful selection of materials and sterilization protocols is crucial to ensuring optimal clinical performance of ACL surgical guides.

## 5. Conclusions

Within the limitations of this study, most of the evaluated resin/3D printer combinations suffered significant dimensional alterations after autoclaving.

## Figures and Tables

**Table 1:** Characteristics and descriptive data of experimental groups.

Group (Technology)	N° SG per Batch (Total)	Printer	Resin	Layer Thickness (µm)	Post-Processing
FL (SLA)	2 (10)	Formlabs Form 3 (Formlabs Inc, Sommerville, MA, USA)	Dental SG (Formlabs. Somerville, MA, USA).	100	Washing and drying protocol using Form Wash (Formlabs, EUA) with 98% isopropyl alcohol for 5 min. Once finished, postpolymerization using Form Cure (Formlabs, USA), during 30 min at 60 °C.
SR (DLP)	2 (10)	SprintRay Pro 95S (SprintRay Inc, Los Angeles, CA, USA)	Surgical Guide 3 (SprintRay. Los Angeles, CA, USA).	100	Washing and drying protocol using ProWash/Dry (SprintRay, USA) with 98% isopropyl alcohol. Once finished, postpolymerization using ProCure (SprintRay, EUA), during 20 min at 50 °C.
ND (DLP)	2 (10)	NextDent 5100 (Vertex Dental B.V., Soesterberg, The Netherlands)	NextDent SG (Vertex Dental B.V., Soesterberg, The Netherlands)	100	Washing and drying protocol using an ultrasonic cleaner with 98% isopropyl alcohol for 5 min. Once finished, postpolymerization using LC-3DPrint Box (3D Systems, Rock Hill, SC, USA), during 10 min at a temperature lower than 45 °C.
KS (LCD)	2 (10)	Phrozen Mighty 4K (Phrozen Tech Co/LTD, Tainan city, Taiwan)	Keyguide (Keystone Industries, Gibbstown, NJ, USA).	100	Washing and drying protocol using an ultrasonic cleaner with 98% isopropyl alcohol for 5 min. Once finished, postpolymerization using Phrozen Cure Mega S (Phrozen Tech Co/LTD, Tainan city, Taiwan) during 30 min at 60 °C.
VC (LCD)	2 (10)	Shining Accufab L4D (Shining 3D, Hangzhou, China)	V Print SG (Voco GmbH, Cuxhaven, Germany)	100	Washing and drying protocol using an ultrasonic cleaner with 98% isopropyl alcohol for 5 min (3 min with reusable alcohol and 2 min with new alcohol). Once finished, postpolymerization using LC-3DPrint Box (3D Systems, USA), during 30 min at 60 °C.

SLA: stereolithography; DLP: digital light processing; LCD: liquid crystal display; FL: Formlabs Form 3/Dental SG; SR: SprintRay Pro 95S/Surgical Guide 3; ND: NextDent 5100/NextDent SG; KS: Phrozen Mighty 4K/Keyguide; VC: Shining Accufab L4D/V Print SG.

**Table 2:** Trueness (RMS) and precision (SD) of the experimental groups at T0 and T1.

Group	Trueness (RMS) (µm)			Precision (SD) (µm)		
	T0	T1	T0 − T1	T0	T1	T0 − T1
FL	81.99 (12.21)	136.56 (35.88)	−54.57 ** **(−72.17 to −36.97)**	81.53 (12.19)	135.87 (35.78)	−54.34 ** **(−71.80 to −36.88)**
SR	138.74 (8.25)	171.13 (17.57)	−32.39 ** **(−49.99 to −14.79)**	137.36 (7.89)	169.70 (16.69)	−32.34 ** **(−49.80 to −14.88)**
ND	107.02 (13.51)	141.95 (18.26)	−34.93 ** **(−52.53 to −17.33)**	104.97 (13.96)	141.19 (18.47)	−36.22 ** **(−53.68 to −18.76)**
KS	110.1 (16.93)	186.78 (28.94)	−76.68 ** **(−94.28 to −59.08)**	109.14 (16.61)	186.22 (29.03)	−77.08 ** **(−94.54 to −59.62)**
VC	107.82 (13.01)	107.84 (16.90)	−0.02 (−17.62 to 17.58)	105.69 (12.07)	105.79 (16.99)	−0.10 (−17.56 to 17.36)

All values at T0 and T1 are reported as mean (standard deviation). T0 − T1 values are reported as mean difference (95% confidence interval). Statistically significant results are in bold. ** (*p* < 0.001). RMS: root mean square; SD: standard deviation; T0: timepoint before autoclaving; T1: timepoint after autoclaving; FL: Formlabs3B+/Dental SG; SR: SprintRay Pro 55S/Surgical Guide 3; ND: NextDent 5100/NextDent SG; KS: Phrozen Mighty 4K/Keyguide; VC: Shining Accufab L4D/V Print SG.

**Table 3:** Inter-group comparative analysis of trueness and precision before sterilization (T0).

T0	Trueness (RMS) (µm)				
Precision (SD) (µm)	FL	56.75 ** **(32.05 to 81.45)**	−25.03 * **(−49.73 to −0.33)**	−28.11 * **(−52.81 to −3.18)**	−25.83 * **(−50.53 to −1.13)**
	55.81 ** **(31.30 to 80.32)**	SR	31.72 * **(7.02 to 56.42)**	28.64 * **(3.94 to 53.34)**	30.92 * **(6.22 to 55.62)**
	−23.44 (−47.93 to 1.05)	32.37 * **(7.86 to 56.88)**	ND	3.08 (−21.62 to 27.78)	−0.80 (−25.50 to 23.88)
	−27.59 * **(−52.09 to −3.09)**	28.21 * **(3.71 to 52.72)**	4.15 (−20.35 to 28.65)	KS	2.28 (−22.42 to 26.98)
	24.20 (−48.71 to 0.32)	31.61 * **(7.11 to 56.11)**	−0.76 (−25.27 to 23.76)	3.40 (−21.11 to 27.90)	VC

Results are the estimates (95% CIs) from the repeated measures mixed-effects model in the cell in common between the column-defining treatment (defined-treatment 1) and the row-defining treatment (defined-treatment 2). Statistically significant results are in bold. * (*p* < 0.05), ** (*p* < 0.001). RMS: root mean square; SD: standard deviation; T1: timepoint after autoclaving; FL: Formlabs3B+/Dental SG; SR: SprintRay Pro 55S/Surgical Guide 3; ND: NextDent 5100/NextDent SG; KS: Phrozen Mighty 4K/Keyguide; VC: Shining Accufab L4D/V Print SG.

**Table 4:** Inter-group comparative analysis of trueness and precision after sterilization (T1).

T1	Trueness (RMS) (µm)				
Precision (SD) (µm)	FL	34.57 * **(9.87 to 59.27)**	−5.39 (−30.09 to 19.31)	−50.22 ** **(−74.92 to −25.52)**	28.72 * **(4.02 to 53.42)**
	33.81 * **(9.30 to 58.32)**	SR	29.18 * **(4.48 to 53.88)**	−15.65 (−40.35 to 9.05)	63.29 ** **(38.59 to 87.99)**
	−5.32 (−29.81 to 19.17)	28.49 * **(3.98 to 53.00)**	ND	44.83 ** **(20.13 to 69.53)**	34.11 * **(9.41 to 58.81)**
	−50.33 ** **(−74.83 to −25.83)**	−16.52 (−41.03 to 7.98)	45.01 ** **(20.51 to 69.51)**	KS	78.94 ** **(54.24 to 103.64)**
	30.05 * **(5.53 to 54.56)**	63.85 ** **(39.35 to 88.35)**	35.37 * **(10.85 to 59.88)**	80.36 ** **(55.87 to 104.88)**	VC

Results are the estimates (95% CIs) from the repeated measures mixed-effects model in the cell in common between the column-defining treatment (defined-treatment 1) and the row-defining treatment (defined-treatment 2). Statistically significant results are in bold. * (*p* < 0.05), ** (*p* < 0.001). RMS: root mean square; SD: standard deviation; T1: timepoint after autoclaving; FL: Formlabs3B+/Dental SG; SR: SprintRay Pro 55S/Surgical Guide 3; ND: NextDent 5100/NextDent SG; KS: Phrozen Mighty 4K/Keyguide; VC: Shining Accufab L4D/V Print SG.

**Table 5:** Axis deviations at the zenith point and area variation between the square-shaped structures from TR to T1.

Group	Axis (mm)			Area (mm^2^)
	X	Y	Z	
FL	0.03 * **(0.01 to 0.06)**	0.06 * **(0.03 to 0.09)**	0.05 (−0.01 to 0.11)	712.82 (712.44 to 713.18)
SR	0.25 * **(0.08 to 0.42)**	0.32 ** **(0.23 to 0.40)**	0.13 * **(0.04 to 0.23)**	710.20 ** **(709.84 to 710.56)**
ND	0.09 (−0.01 to 0.19)	0.13 * **(0.06 to 0.20)**	0.03 (−0.01 to 0.07)	712.73 (712.37 to 713.08)
KS	0.04 * **(0.01 to 0.06)**	0.10 * **(0.05 to 0.15)**	0.10 * **(0.02 to 0.19)**	712.73 (712.22 to 713.25)
VC	0.03 ** **(0.02 to 0.04)**	0.09 ** **(0.07 to 0.11)**	0.11 * **(0.04 to 0.18)**	712.73 (712.60 to 712.86)

All values are reported as Mean (95% Confidence interval). Statistically significant results between TR and T1 are in bold. * (*p* < 0.05), ** (*p* < 0.001). FL: Formlabs3B+/Dental SG; SR: SprintRay Pro 55S/Surgical Guide 3; ND: NextDent 5100/NextDent SG; KS: Phrozen Mighty 4K/Keyguide; VC: Shining Accufab L4D/V Print SG.

## Data Availability

The original data presented in this study are openly available in the CORA repository at https://doi.org/10.34810/data2447.

## Abbreviations

The following abbreviations are used in this manuscript:

SG	Surgical guide
ACL	Aesthetic crown lengthening
CAD/CAM	Computer-aided design and computer-aided manufacturing
SM	Subtractive method
AM	Additive method
APE	Altered passive eruption
SLA	Stereolithography
DLP	Digital light processing
LCD	Liquid crystal display
CLIP	Continuous liquid interface production
CBCT	Cone beam computerized tomography
DICOM	Digital imaging and communication in medicine
STL	Standard Tessellation Language
ROI	Region of interest
SD	Standard deviation
RMS	Root mean square

## Appendix A

**Table A1:** Chemical composition of the resins employed in this study.

Group	Composition	%	CAS Nº
FL	BIS-EMA (Poly(oxy-1,2-ethanediyl), α,α’-[(1-methylethylidene)di-4,1-phenylene]bis[ω-[(2-methyl-1-oxo-2-propenyl)oxy]-)	≥75%	41637-38-1
	Urethane dimethacrylate (7,7,9(or 7,9,9)-trimethyl-4,13-dioxo-3,14-dioxa-5,12-diazahexadecane-1,16-diyl bismethacrylate)	30–50%	72869-86-4
	phenyl bis(2,4,6-trimethylbenzoyl)-phosphine oxide	<10%	162881-26-7
SR	BIS-EMA (Ethoxylated bisphenol a dimethacrylate)	30–70%	41637-38-1
	Trimethylolpropane formal acrylate	30–70%	66492-51-1
	Urethane dimethacrylate	15–35%	72869-86-4
	TPO (Diphenyl(2,4,6-trimethylbenzoyl) phosphine oxide)	≤5	75980-60-8
	Trimethylolpropane triacrylate	≤5	15625-89-5
ND	BIS-EMA (Bisphenol A Polyethylene Glycol Diether Dimethacrylate)	≥75%	41637-38-1
	Urethane dimethacrylate (7,7,9(or 7,9,9)-trimethyl-4,13-dioxo-3,14-dioxa-5,12-diazahexadecane-1,16-diyl bismethacrylate)	10–20%	72869-86-4
	phenyl bis(2,4,6-trimethylbenzoyl)-phosphine oxide	1–5%	162881-26-7
KS	2-hydroxyethyl methacrylate	≥10 to ≤25%	868-77-9
	Isobornyl methacrylate	≥10 to ≤25%	7534-94-3
	TPO (Diphenyl(2,4,6-trimethylbenzoyl) phosphine oxide)	<3%	75980-60-8
VC	BIS-EMA	50–100%	41637-38-1
	Urethane dimethacrylate	10–25%	72869-86-4
	TPO (Diphenyl(2,4,6- trimethylbenzoyl)phosphine oxide)	≤2.5%	75980-60-8
